# Field Investigation of Clay Balls in Full-Depth Asphalt Pavement

**DOI:** 10.3390/ma12182879

**Published:** 2019-09-06

**Authors:** Weiguang Zhang, Jusang Lee, Hyung Jun Ahn, Qiqi Le, Meng Wu, Haoran Zhu, Jing Zhang

**Affiliations:** 1 School of Transportation, Southeast University, Nanjing 211189, China (Q.L.) (M.W.) (J.Z.); 2 Indiana Department of Transportation, West Lafayette, IN 47906, USA; 3 Virginia Department of Transportation, Richmond, VA 23219, USA; 4 JSTI Group, Nanjing 210019, China

**Keywords:** clay ball, asphalt pavement, pattern and density, infrared image collection system, field core

## Abstract

Clay ball is a pavement surface defect which refers to a clump in which clay or dirt is mixed with hot asphalt mixture. Clay ball is typically caused by a combination of aggregate contamination of clay or soil, high aggregate moisture, and low production temperature at the asphalt plant. It usually appears a few weeks or months after paving under traffic load, after being liquefied and knocked from the pavement surface. Clay balls can be the source of potholing, raveling, and other issues such as moisture infiltration and reduced ride quality. This paper presents an investigation of the clay balls on US-31 one winter after construction in Hamilton County, Indiana. In order to understand the pavement condition, their severity was measured using both visual observation and infrared image collection system. In addition, a clay ball amount, its distribution pattern, and cores condition were evaluated. A precipitation effect on clay ball formation was investigated for finding a cause of the clay balls. The investigation found that infrared image collection system was appropriate in detecting the clay balls. The clay balls were elliptic in shape with 2.5 cm to 10 cm in diameter, and the maximum clay ball depth was almost penetrating the entire surface course. It was also found that the asphalt paving on the raining days or right after raining could increase the potential of clay balls. Monitoring of aggregate moisture during construction on or after raining days should be able to reduce the risk of clay balls.

## 1. Introduction

Clay ball, also called dust ball or dust cake, is a pavement surface defect in both asphalt pavement and Portland Cement Concrete pavement. It refers to a clump in which clay or dirt is mixed with hot asphalt mixture. Clay ball may be well coated by asphalt during mixing and compaction process, while no asphalt coats on the exposed surface are found when the ball is partially sliced [1]. A clay ball is small in size and can either appear near pavement surface or inches below pavement surface [2]. A clay ball usually appears a few weeks or months after paving, but some may not appear until a full winter season of freeze-thaw cycles has occurred [3,4].

A clay ball could initiate from aggregate contamination of clay or soil before arriving on the plant site or aggregate pollution from the soil below stockpile. The clay or dirt mixes into the hot asphalt mixture with the coarse or fine aggregates at a drum and creates clumps. A clay ball could also initiate from a combination of moisture in the aggregate and lower temperatures during mixing, then fines can “cake” on the mixing paddles in the drum. In this case, lower temperatures may not be adequate to cook out the moisture in the aggregate. The fines collect with the moisture and stick to these paddles. These clumps will be placed and compacted in the field road section together with uniform hot mix asphalt. At first, clay balls may or may not be evident. Over time, voids may be generated by fine particles absorbing water and expanding when frozen to cause a void at the surface (or very near the surface), or if traffic loadings break or crack the thin mortar-skin above the clump to expose the clay ball. The clay ball floats up to the pavement surface during traffic load since they weigh less than the aggregates and other particles in the surrounding mixture.

Several other activities could increase the possibility of clay ball initiation. For instance, central asphalt mix plants are usually more susceptible to producing clay balls, simply because they are temporarily placed on right-of-way near the project site which may have clay or loose soil underneath the stockpiles [3]. Moreover, the loader operator must apply the right amount of down pressure on the loader blade of the front-end loader. Too much pressure or too sharp an angle will cause the front blade to scrape the soil beneath the stockpile, thereby picking up the mud or clay, which will introduce soil particles into the mixture. Thus, the stockpile management should be carefully conducted to avoid the clay ball during field construction. 

Scullion and Harris [2] analyzed the components of clay ball based on field cores taken from cement treated base layer. An X-ray diffraction (XRD) analysis was performed, and results show that the clay balls were predominantly smectite, mica, kaolinite, quartz, and calcite. Some of the clay balls have high silicon and aluminum concentrations typically of aluminosilicate minerals. 

The existence of clay ball could deteriorate pavement. For instance, the areas with clay ball may not be stable with a source of water over time. With water, these clay balls are liquefied, eroded, or knocked from the surface by traffic load and eventually are the source of potholing and raveling [1,5,6,7]. Additional damages that could be caused by clay balls including moisture infiltration and reduced ride quality. It is believed that with proper repair, clay balls do not cause a long-term performance issue, such as structure related distresses [8,9]. 

In 2012, a number of clay balls were observed after the first winter on US-31 in Hamilton County, Indiana. The clay ball was not widely seen in Indiana previously, and it is not sure how severe they are and if the pavement performance could be negatively affected accordingly. Thus, Indiana Department of Transportation (INDOT) conducted a comprehensive evaluation with extensive forensic testing to determine the cause of such distress type. The pattern and density of these clay balls were checked as well. The “pattern” here indicates how the clay balls were located along pavement longitudinal direction (parallel to traffic), and distribution comparison among driving lane, shoulder and ramp. An infrared imaging system was also used to detect clay ball at traffic speed to see if this technology matched well with visual observation. Cores were taken from the clay ball areas and the size (diameter and depth) of each clay ball was measured. In addition, mix design, traffic, and climatic data were also collected to check if they were correlated with clay ball pattern or density as suggested elsewhere [10,11,12].

The objectives of this study were to (a) evaluate the accuracy of infrared image collection system in detecting position of clay balls; (b) determine the pattern and density distribution of clay balls in the field, and (c) determine the potential factors that could be correlated with clay ball pattern. 

## 2. Materials and Methods 

A high-speed infrared image collection system was used to detect the location of exposed clay balls. Distance measuring instrument (DMI) system and the Global Positioning System (GPS) were installed to obtain the accurate spatial location of each clay ball. The detection results were further confirmed by visual observation performed by workers who were responsible for clay ball repair. 

The pattern of the clay ball was analyzed. In specific, a clay ball distribution along the longitudinal direction (parallel to traffic) was plotted to characterize its pattern, such as if they were distributed evenly or concentrated within specific areas, or if they were spread with fixed distance. The distribution of clay ball within the driving lane, passing lane, shoulder, and ramp was also compared. 

Cores with visually observed clay balls were taken from pavement surface course, which was collected to check their shape, diameter, and depth by slicing the core in the middle of popout. Cores without visually observed clay ball were also obtained to verify if there were any existing clay balls not exposed to pavement surface. Such inspection was necessary since the existed clay ball not exposed to the surface could be the potential weak point. In the laboratory, such cores were sliced into multiple pieces to examine the existence of clay ball. 

One winter after the clay ball was repaired, the researchers re-visited the clay ball areas to check if there were any newly developed clay balls or if the previously repairing of clay balls worked properly. 

The construction information, such as mix design and truck coverage paving length, as well as climatic information, was collected to determine which parameter(s) could be potentially correlated with clay ball commencement and distribution pattern. 

### 2.1. Pavement Condition

The $19.6 million interchange improvements and roadway reconstruction at the intersection of US-31 (two lanes in both directions) and SR 38 in Hamilton County, Indiana, were built in summer 2012. The amount of hot mix asphalt (HMA) was 63,818 tons for mainline, shoulders and ramps. The Average Daily Truck Traffic (AADT) was recorded as 24,814 in 2011. 

**Figure 1:**
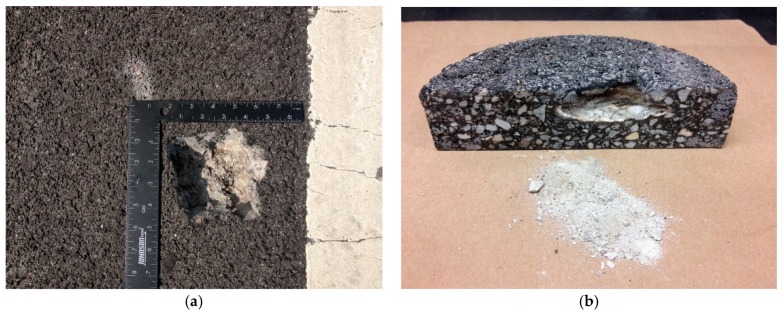
Field observation of clay ball. (**a**) Opened and cleaned clay ball; (**b**) sliced core from clay ball area.

In spring 2013, the interchange pavement exhibited 633 popouts from the surface course in driving lanes, shoulder, and ramps. These popouts are small size holes with fine particles surrounding the holes. Referring to the repair method used in another project constructed in 2001, the clay balls in this project were repaired by cleaning and sealing with hot poured asphalt sealant individually. Figure 1a shows a clay ball after opened by a jack hammer and cleaned by a vacuum. Figure 1b is a field core from clay ball area after slicing in the middle of the hole. Over time, these clay balls which appeared to be agglomerations of fine material disintegrate from weather and leave a hole in the mix after being washed out.

### 2.2. Materials and Construction

The pavement type was full-depth HMA pavement with design surface thickness of 5.1 cm and total HMA thickness of 30 cm. The asphalt treated base was constructed between subgrade and asphalt layers. Two types of asphalt mix were used in the project. In the main lanes and ramps, a 9.5 mm nominal maximum aggregate size (NMAS) mix with PG76-22 binder was used for equivalent single axel load (ESAL) category 4. In the shoulder, a 9.5 mm NMAS mix was used with PG 64-22 binder corresponds to ESAL category 1. 

The job mix formula (JMF) had been used for other projects with no problem. The aggregate source had been used for more than forty years without any issue. Table 1 summarizes aggregate type and percentage of each aggregate source. An aggregate drum mix dryer with a maximum capacity of 600 tons per hour, equipped with a natural gas fired aggregate dryer burner with a maximum rated power of 200 million British thermal units per hour (58.6 million watts) was used for HMA production. In general, the measured plant temperatures (159 °C to 176 °C) during construction met the INDOT specifications [13]. The paving lasted fourteen days, started on 12 October 2012 and ended on 25 October 2012. 

Samples were collected and tested for a Quality Assurance (QA) purpose as shown in Table 2. INDOT QA factors for HMA production include binder content, air voids, and voids in mineral aggregate (VMA). Overall HMA QA pay factors were 1.02 and 1.035 for main lane and shoulder mix, respectively, which indicates that the HMA exceeded average quality (pay factor equals to 1.0). Another INDOT HMA construction QA factor, the percentage of density, was also listed in Table 2. Overlay, pay factor for the density, were higher than 1.0 except one lot with main lane construction (with 0.94 pay factor). 

### 2.3. Field Core

**Figure 2:**
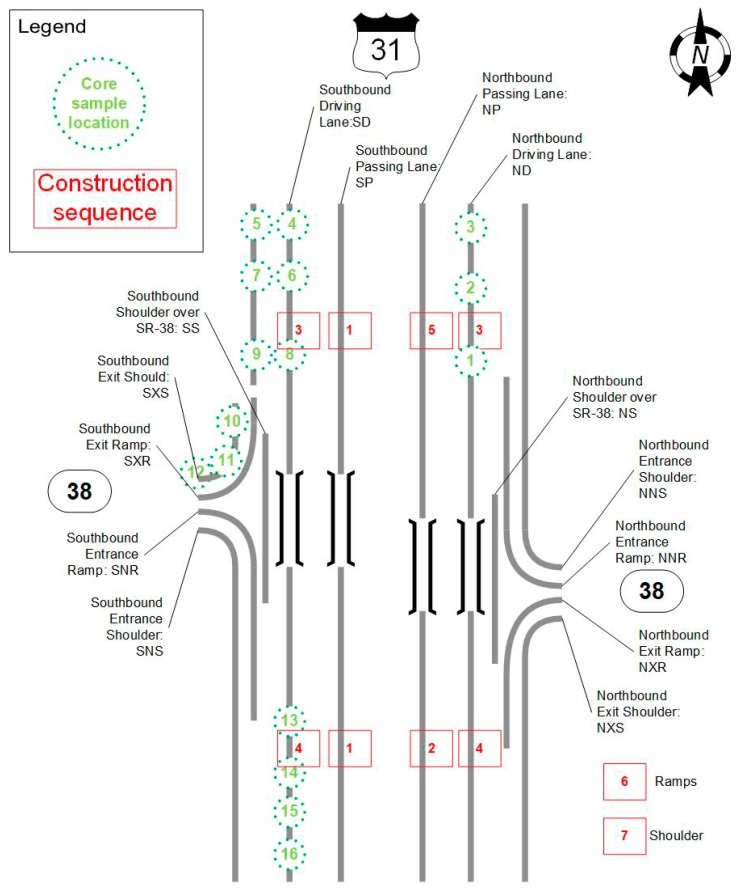
Schematic of US-31 and SR-38 with core location and construction sequence.

Two types of cores were taken: cores with clay balls and cores without clay ball from good and bad sections based on visual observation. Popouts were only observed at pavement surface, and thus the coring depth was 2 inches, which covered the entire surface course depth. These cores were drilled on 12 August 2013 and were delivered back to the laboratory for detailed inspection. Figure 2 presents a schematic plot that shows the project layout with core location and construction sequence. As shown, the cores were taken from paving sections with low frequencies of clay balls, such as core sample locations 4, 6, 8, and 13 to 16, as well as from paving sections with high frequencies of clay balls, such as core sample locations 5, 7, 9, 10, and 11. Cores were taken from driving lane, passing lane, shoulder, and ramp. Note that a couple of samples were taken within each core location, and a total of 633 cores were obtained. 

### 2.4. Infrared Image Collection System

**Figure 3:**
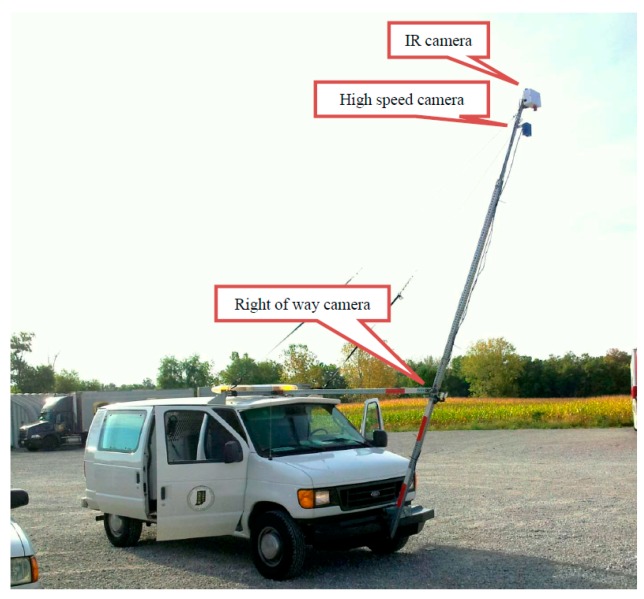
Infrared image collection system.

The detection of clay balls can be achieved by measuring the density or texture of asphalt pavement. Pavement sections with observed clay balls are usually characterized with extreme high in-place air voids or very non-uniform texture distribution [14,15]. In this study, in order to rapidly detect clay balls, pavement surface images were collected and analyzed using the INDOT pavement infrared image collection system integrated with an infrared (IR) camera, a gray scale high-speed camera and a right of way camera (iPhone 4S) as shown in Figure 3. The system consists of a van, three cameras that were fixed to a bar, and a computer to acquire image data. As shown, the IR camera was placed at the very top of the bar and took photos that were perpendicular to the pavement in vertical direction. The gray scale high-speed camera was set in the middle. The right of way camera was put close to pavement surface which was used to capture clay ball. A distance measuring instrument (DMI) system was used to measure the distance the van drove. The IR camera was triggered at a fixed distance based on DMI data. The right of way camera embedded an iPhone 4S cell phone to take photos and used GPS to determine location. Both cameras covered the width of one entire traffic lane. The operation speed ranged between 5 and 10 mph. The image collection was conducted from 9:30 to 15:30 on 17 September 2013. Two replicates were conducted in the morning and in the afternoon, respectively to avoid any potential test error.

## 3. Results and Discussion

### 3.1. Infrared Image Collection System

**Figure 4:**
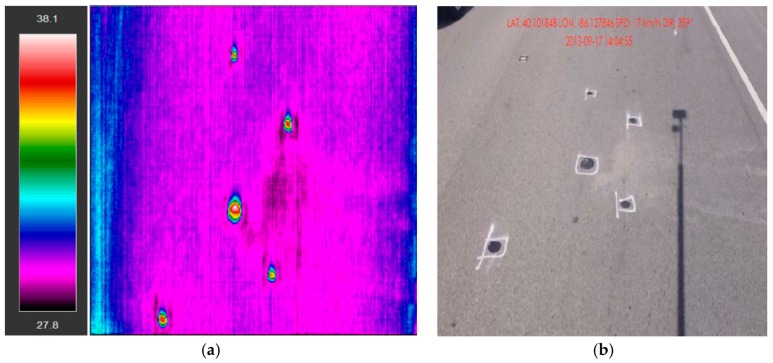
Clay ball images from (**a**) IR camera and (**b**) right way of camera.

Figure 4a,b shows the images taken from the IR camera and the right of way camera, respectively. Note that the two cameras were taking photos simultaneously. As shown, all the five popout locations in Figure 4b were successfully captured by the IR camera in Figure 4a. The different colors in the IR image indicate temperature differentiation. The clay balls were identified in these areas with high temperature differentiate (i.e., higher temperature in the middle with lower temperature around). In general, a total of 633 holes (346 holes on the mainline and shoulders and 287 holes from ramps) were cleaned and filled with asphalt sealant, among which 564 cores were successfully captured by the IR camera. Among all the clay balls (69 in total) that were not successfully captured by the IR, the majority (50 in total) were missed due to the very small size of clay balls (i.e., with diameter less than 1 cm). It is noted that the angle of the two photos are not exactly the same: The IR camera took photos vertically perpendicular to pavement surface, whereas the right of way camera took photos with an angle to pavement surface.

### 3.2. Distribution of Clay Ball

A few small voids caused by clay balls can be ignored as a byproducts of the heavy construction process, but many significant voids in the pavement surface warrants investigation, such as the case in this study. Table 3 shows the amount of clay balls based on each paving sequence. The construction date is also presented. As seen, the amount of clay ball varies greatly from one day to another. The highest clay ball every 61 m was 2.82 from shoulder area (paving sequence 7), whereas the lowest clay ball very 61 m was 0.49 from passing lane area (paving sequence 1). 

**Figure 5:**
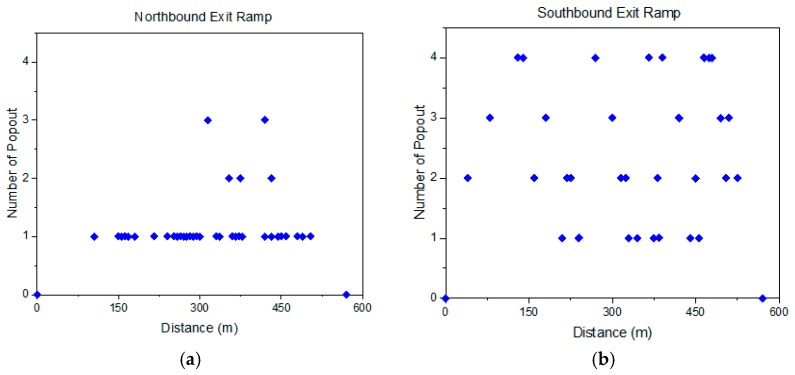

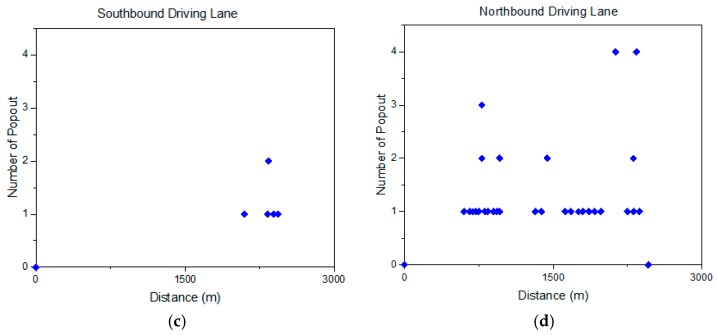
Clay ball distribution within (**a**) southbound exit ramp, (**b**) northbound exit ramp, (**c**) southbound driving lane, and (**d**) northbound driving lane.

Figure 5 presents the clay ball distribution in the longitudinal direction (parallel to the direction of vehicle traffic) for both driving lane and ramp. The majority of the popouts were found in shoulder, ramp, and various locations in the driving lanes, shoulder, and ramp but few in the passing lanes. Additionally, most popouts were from the pavement location between wheel paths, whereas very few of these pop-out locations were witnessed within the wheel paths. 

As shown in Figure 5, the number of popout within southbound exit ramp is almost evenly distributed with around 61 m to 73 m as the gap distance. It should be noted that the typical laydown lengths of 20-ton surface mix in a truck with 165 lbs/yd^2^ rate were 55 mt for main lane (3.7 m width) and 66.4 m for shoulder (3 m width). Thus, it is possible that the non-uniform mix between two trucks could be a reason that caused clay ball in this area. 

However, such trend was not observed in northbound exit ramp or driving lane. In those areas, the clay balls were distributed either as close as several meters and as far as one hundred meters. The fact that the “pop-out” locations do not seem to be consistent throughout the mix and the fact that these areas seem to be confined to what would amount to a typical truckload of material at a time based on length and concentration of the pop-outs and the randomness of these locations should rule out the possibility of the paving operation being an issue.

### 3.3. Size and Shape of Clay Ball

The depth, size, and shape are major parameters to characterize a clay ball [16,17,18,19,20] and were measured. The six-inch core samples taken from popout locations were cut to reveal a rectangular cross-section. All the sliced cores were taken between or away from the wheel-path to avoid the effect of trafficking compaction. In order to have a side view of clay ball, cores were cut in the middle of a clay ball opening hole using a diamond coated wire saw. The typical circular saw used to cut the asphalt core had a 3.3 mm thick saw bit. Furthermore, the lateral movement of the saw bit in cutting operation may have caused a wider sample thickness loss. To overcome this limitation, a diamond-impregnated wire saw was utilized. This saw is a customized product designed to make a series of programmed cuts for the sample cores. The diameter of the diamond-impregnated wire was only 0.2 mm, with very small lateral movements due to fixed pulleys in the saw system. The samples were dry-cut to keep the clay ball inside intact.

**Figure 6:**
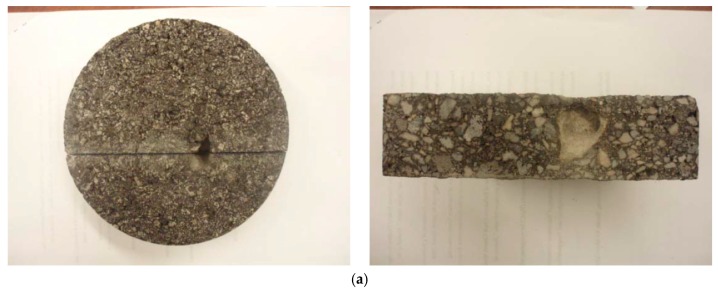

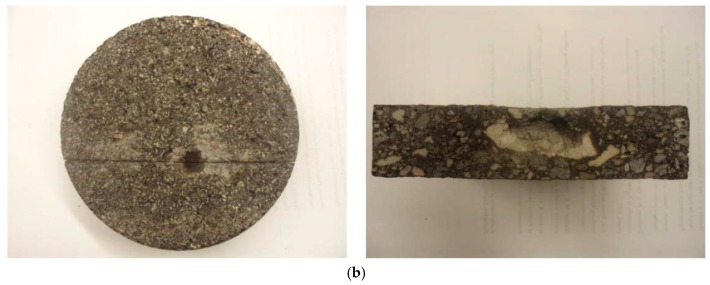
Clay balls with (**a**) one inch in diameter and (**b**) two inches in diameter.

Figure 6 shows two clay ball examples that were squeezed, and their openings were collapsed. The left figure was pictured upside down, and the right figure shows sliced core in the middle of the hole. The diameter of each clay ball was measured and was ranged from 1 to 4 inches in diameter. Considering the ellipse sphere shape of the clay ball, several measurements were conducted and averaged. As seen, the hole on pavement surface is small in scale but the clay ball size is much bigger than the hole. The depth of the clay ball almost punctures the entire surface course thickness of 2 inches. It is also noted that good amounts of fines remained in the voids.

### 3.4. Cores Without Visually Observed Popouts

**Figure 7:**
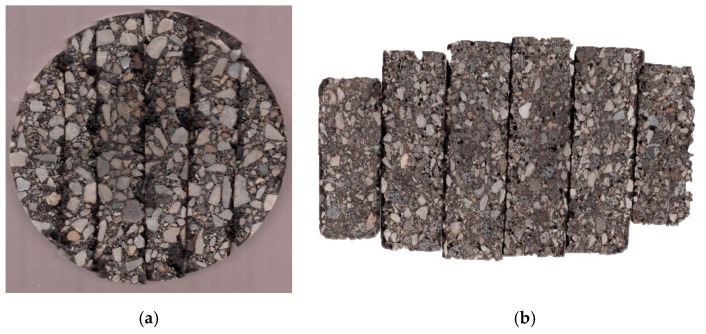
Cut surface of core sample; (**a**) top-down view and (**b**) inside of core.

As aforementioned, a couple of cores were taken from pavement areas without clay balls to verify if there was any clay ball not exposed to the pavement surface. In the laboratory, each six-inch core was sliced into several pieces. Figure 7a,b shows an example of sliced core from top and side, respectively. As seen in Figure 7b, the samples are very clean and no clay ball was found. Additionally, there was no aggregate size larger than 1 inch in diameter, the critical size of clay ball for the popout. All the rest cores were sliced and no clay balls were observed as well, even for the cores taken from bad sections (with the large frequency of clay balls). This indicates that in the project evaluated all the clay balls were exposed to the pavement surface.

### 3.5. Relationship between Moisture and Clay Ball

**Figure 8:**
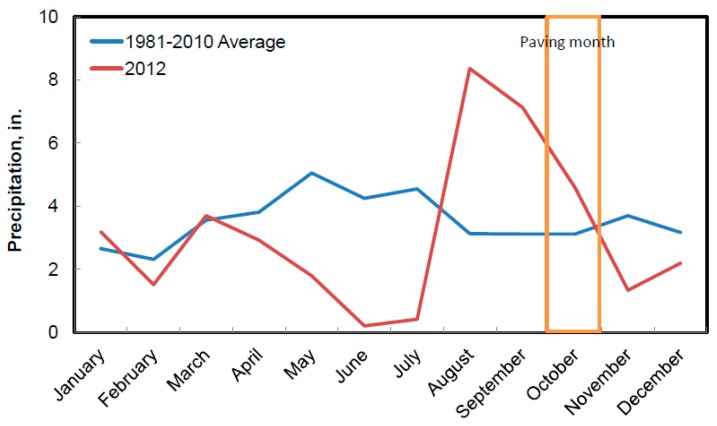
Comparison of monthly precipitations.

During paving, there were seven raining days, and no construction was conducted on those days. Figure 8 shows precipitation distribution within each month in 2012, as well as the average monthly precipitation based on years from 1981 to 2010. It is shown in the figure that precipitations in October 2012 were much high compared to its companion averaged from 1981 to 2010. 

**Figure 9:**
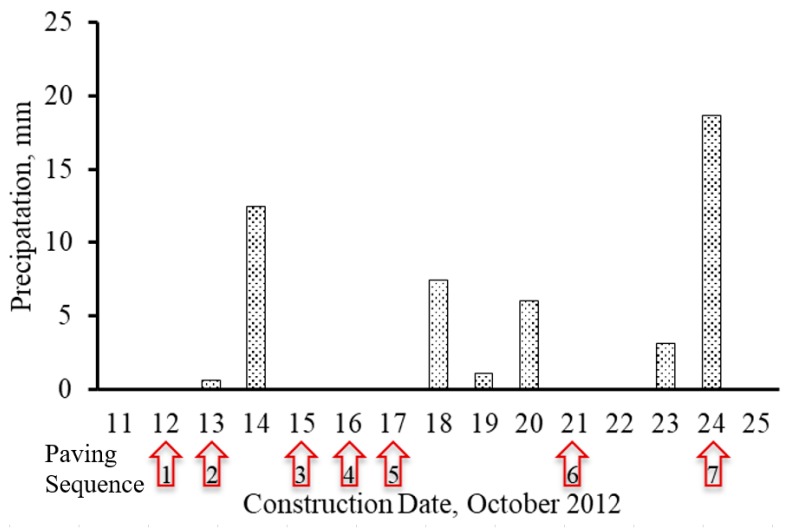
Comparison of daily precipitations to paving dates.

Figure 9 presents the precipitation during the days the road was paved with the paving sequences 1 to 7. As shown, the more precipitation could result in more moisture in aggregate stockpiles, and the wet aggregates may cause the material to become sticky and bind together, which increases the possibility of clay balls in a drum mixer. In general, paving in a day after the rain showed higher clay ball densities than others. For instance, paving sequences 3 and 7 were constructed one day after raining and showed the highest clay ball density. It is noted that typically aggregate moisture was checked before construction, while raining during paving days could greatly increase aggregate moisture and increase the possibility of clay balls. Thus, check for aggregate moisture during construction, especially on or after raining could be necessary. 

### 3.6. Revisit of Pavement Sections with Clay Ball

After two winters, the pavement section was revisited. It was found that the number of clay balls did not increase, which means that no new clay balls were developed. The patching worked very well without loss of materials. This indicates that clay balls should be a one-time distress and may not affect the long-term field performance of pavement as long as it is well repaired.

## 4. Conclusions

This study evaluated the clay balls that were observed on US-31 in Hamilton County, Indiana. The detection of clay ball using infrared image collection system was evaluated. The clay ball amount and distribution pattern were also checked. In addition, field cores were taken and the depth and diameter of clay balls were measured. The cores without visually observed popouts were also collected to see if any clay balls were not detected. Precipitation information during construction was also collected to check the potential relationship between field moisture and clay ball density. Based on the analysis, the following conclusions can be drawn:

- Based on engineering judgement, the infrared image collection system was found to be accurate enough (detection accuracy of 564 out of 633) in pinning the clay ball location.
- Regarding cores with clay balls, the clay ball diameter ranged from 2.5 cm to 10 cm, and the maximum clay ball depth is almost penetrating the entire course’s surface. Most clay balls were elliptic in shape.
- The asphalt paving on raining days or right after raining could increase the potential of clay balls. It is recommended to check the aggregate moisture right after or on raining days and take the necessary steps such as extended mixing time to reduce the risk of clay balls.

## Figures and Tables

**Table 1:** Summary of raw aggregate and recycled asphalt pavement (RAP).

Aggregate Type	Main Lane, %	Shoulder, %
Dolomite	24.0	30.0
Blast furnace slag	20.8	-
Dolomite sand	28.0	-
Stone sand	10.0	24.0
RAP	16.0	45.0
Baghouse fines	1.2	1.0
Aggregate total	100.0	100.0

**Table 2:** Summary of Quality Assurance (QA) test results.

Parameter	Main Lane	Shoulder
Binder, %	6.85	5.65
Air voids, %	4.56	4.35
VMA, %	15.58	16.3
Density, %	92.57	93.25

**Table 3:** Summary of project information.

Paving Sequence	Date	Length, m	Number of Clay Balls	Clay Ball Every 61 m
1	10/12/2012	2631	21	0.49
2	10/13/2012	1564	14	0.55
3	10/15/2012	2134	79	2.26
4	10/16/2012	3136	76	1.48
5	10/17/2012	762	10	0.80
6	10/21/2012	3039	53	1.05
7	10/24/2012	6935	321	2.82
